# CoREST1 Promotes Tumor Formation and Tumor Stroma Interactions in a Mouse Model of Breast Cancer

**DOI:** 10.1371/journal.pone.0121281

**Published:** 2015-03-20

**Authors:** Sohini Mazumdar, Lisa M. Arendt, Sarah Phillips, Maja Sedic, Charlotte Kuperwasser, Grace Gill

**Affiliations:** 1 Department of Developmental, Molecular and Chemical Biology, Sackler School of Graduate Biomedical Sciences, Tufts University School of Medicine, Boston, Massachusetts, United States of America; 2 Genetics Program, Sackler School of Graduate Biomedical Sciences, Tufts University School of Medicine, Boston, Massachusetts, United States of America; 3 Cellular, Molecular and Developmental Biology Program, Sackler School of Biomedical Sciences, Tufts University School of Medicine, Boston, Massachusetts, United States of America; 4 Molecular Oncology Research Institute, Tufts Medical Center, Boston, Massachusetts, United States of America; University of South Alabama, UNITED STATES

## Abstract

Regulators of chromatin structure and gene expression contribute to tumor formation and progression. The co-repressor CoREST1 regulates the localization and activity of associated histone modifying enzymes including lysine specific demethylase 1 (LSD1) and histone deacetylase 1 (HDAC1). Although several CoREST1 associated proteins have been reported to enhance breast cancer progression, the role of CoREST1 in breast cancer is currently unclear. Here we report that knockdown of CoREST1 in the basal-type breast cancer cell line, MDA-MB-231, led to significantly reduced incidence and diminished size of tumors compared to controls in mouse xenograft studies. Notably, CoREST1-depleted cells gave rise to tumors with a marked decrease in angiogenesis. CoREST1 knockdown led to a decrease in secreted angiogenic and inflammatory factors, and mRNA analysis suggests that CoREST1 promotes expression of genes related to angiogenesis and inflammation including VEGF-A and CCL2. CoREST1 knockdown decreased the ability of MDA-MB-231 conditioned media to promote endothelial cell tube formation and migration. Further, tumors derived from CoREST1-depleted cells had reduced macrophage infiltration and the secretome of CoREST1 knockdown cells was deficient in promoting macrophage migration and macrophage-mediated angiogenesis. Taken together, these findings reveal that the epigenetic regulator CoREST1 promotes tumorigenesis in a breast cancer model at least in part through regulation of gene expression patterns in tumor cells that have profound non-cell autonomous effects on endothelial and inflammatory cells in the tumor microenvironment.

## Introduction

Breast cancers originate from epithelial cells which have sustained mutations in oncogenes leading to dysregulated proliferation [1]. In order to progress to malignancy, growing tumors need to form new blood vessels within the local microenvironment in order to acquire nutrients and oxygen and get rid of metabolic waste. Multiple cell types in the tumor parenchyma have been implicated in the promotion of angiogenesis, including the developing tumor cells, invading immune cells, and cancer associated fibroblasts (for review, [2–5]). Developing tumors secrete an abundance of inflammatory regulators including growth factors and cytokines, which recruit and activate immune cells, including tumor associated macrophages (TAMs). TAMs, in turn, produce a number of cytokines and proteases that affect endothelial, epithelial and mesenchymal cells in the tumor microenvironment [6,7]. The complex network of factors that allow a developing tumor to activate the local microenvironment leading to angiogenesis is incompletely understood.

Enzymes that post-translationally modify histones and other chromatin associated proteins play important roles in regulating transcription programs that dictate cell fate, and aberrant activity of these enzymes can contribute to tumor initiation and progression [8]. For example, since histone acetylation is generally associated with actively transcribed genes, increased activity of histone deacetylases (HDACs) may contribute to aberrant gene silencing in tumors. HDAC1 and 2 are often overexpressed in cancer and frequently correlate with poor prognosis, although high levels of HDAC1 in breast cancer have been correlated with better outcomes [9]. The lysine-specific demethylase LSD1 (KDM1A) has also been associated with transcriptional repression via demethylation of mono- and di-methlylated H3K4 [10,11]. LSD1 levels have been found to be elevated in multiple tumor types, including breast cancer [8,12–14]. Data from both *in vitro* and *in vivo* models support a role for LSD1 in the promotion of breast tumor growth [13,15–18]. In particular, high levels of LSD1 have been correlated with aggressive, ER negative, basal-type breast cancers [16]. Inhibitors targeting HDACs or LSD1 are promising therapeutic candidates under active investigation.

Interestingly, the CoREST1 corepressor is intimately associated with LSD1 and is found in large CoREST1/LSD1/HDAC1/2 corepressor complexes [10,19,20]. CoREST1 was originally discovered as a corepressor for the transcription factor, REST, although additional transcription factors, and possibly lncRNAs, also recruit CoREST1/LSD1 [21–27]. CoREST1 regulates the recruitment and activity of associated deacetylase and demethylase enzymes; *in vitro*, CoREST1 is required for HDAC1 and LSD1 activity on nucleosomes [10,28,29]. Although most well described for its corepressor functions, the LSD1/CoREST1 complex has also been suggested to activate transcription in some contexts, through demethylation of alternate substrates (other than H3K4) [30,31]. Despite the fact that histone modifying enzymes associated with CoREST1 have been implicated in cancer, the role of CoREST1 in breast cancer progression has not been characterized.

Here we investigated the role of CoREST1 in the growth and progression of basal-type breast tumors. We examined the effect of CoREST1 depletion in MDA-MB-231 breast cancer cells on tumor formation in xenograft studies as well as on the effects of tumor cell conditioned media on endothelial cells and macrophages in culture. Our data demonstrate that CoREST1 regulates levels of secreted angiogenic and inflammatory factors to impact angiogenesis and tumor-induced inflammatory responses. Our findings implicate a necessary role for CoREST1 in tumor angiogenesis and reveal the importance of CoREST1 in tumor/stroma interactions including the recruitment of TAMs.

## Materials and Methods

### Cell lines and tissue culture

MDA-MB-231 and 293T cells were grown in Dulbecco's Modified Eagle's Media (DMEM; Invitrogen) supplemented with 10% fetal bovine serum (FBS). SUM159 cells were grown in Ham’s/F12 media (Invitrogen) supplemented with insulin (5mg/mL), hydrocortisone (0.5mg/mL), and 5% calf serum. HL-60 cells were grown in RPMI 1640 (Invitrogen) supplemented with 10% FBS and 50mM HEPES. All cell lines tested negative for mycoplasma (MilliPROBE; Millipore); however the identity of each cell line was not authenticated in our laboratory. Human umbilical vascular endothelial cells (HUVEC) obtained from Lonza were grown in the endothelial growth media bullet kit (Lonza). HL-60 cells were differentiated into adherent macrophages as previously described [32].

MISSIONshRNA targeting CoREST1 were obtained from Sigma (#1 TRCN0000147958, #2 TRCN0000418894). MISSION pLKO.1-puro Non-Target shRNA was used as a control (shCtrl; Sigma). Lentivirus was generated as previously described [33]. Briefly, lentiviral particles were generated by cotransfection of the shRNA construct with pCMV-VSVG, expressing the vesicular stomatitis virus glycoprotein and the packaging construct pCMVΔR8.2Δvpr into 293T cells with the FuGENE 6 transfection reagent (Promega). Lentivirus-containing supernatant from the transfected 293T cells was filtered through a 0.45μm syringe filter and used to directly infect subconfluent MDA-MB-231 cells in the presence of 5μg/mL protamine sulfate (Sigma). shCoREST1 and shCtrl cells with lentiviral integration were selected with 1μg/mL puromycin. An average of 70% of the cells from each infection survived selection.

### Animals

The care of animals and all animal procedures were conducted in accordance with a protocol approved by the Tufts University IACUC committee. The Animal Welfare Assurance Number is A-3775-01. Mice were given food and water *ad libitum*. MDA-MB-231 cells were trypsinized (0.05%, Invitrogen), counted and resuspended in a 1:1 dilution of Matrigel (BD Biosciences) and cell growth media. 1x10^6^ cells were injected in 30μl into the fourth mammary fat pad of 8 week old NOD/SCID female mice (Jackson Laboratories). All surgeries were performed under isofluorane anesthesia, and all efforts were made to minimize suffering. Tumors were measured twice weekly with calipers, and mice were humanely euthanized using CO_2_ asphyxiation followed by cervical dislocation when tumors reached 1.5 cm in diameter. Cell growth greater than 3 mm in diameter was considered a tumor. At the time of tissue collection, tumors were weighed, and a portion was fixed in formalin for histology or frozen for molecular analyses.

### Western blotting

Cells were resuspended in RIPA buffer supplemented with protease cocktail inhibitors (Roche, cOMPLETE EDTA free) and incubated on ice for 30 min with intermittent vortexing. Lysates were passed through Qiashredder spin columns (Qiagen), and protein was quantified using the DC Protein Assay (Bio-Rad). Immunoblots were incubated overnight at 4°C with rabbit anti-human polyclonal CoREST1 (1:2500; Millipore; cat. no. 07-455), rabbit anti-human monoclonal LSD1 (1:1000; Cell Signaling Technology; cat. no. 2184), mouse anti-human monoclonal β-actin (1:10,000; Abcam; cat. no. ab6276) or mouse anti-rabbit monoclonal GAPDH (1:10,000; Millipore; cat. no. MAB374) diluted in 5% bovine serum albumin (BSA) or milk in TBST. Goat anti-rabbit or anti-mouse secondary antibodies (1:5,000; Cell Signaling Technologies; anti-mouse 70745; anti-rabbit 70765) were applied for 1 hr at room temperature.

### Quantitative PCR analyses

RNA was isolated utilizing TRIzol (Invitrogen) or QIAzol (Qiagen) according to the manufacturers’ protocols. RNA samples were reverse transcribed using iScript cDNA kit (Bio-Rad), and quantitative PCR (qPCR) was performed with SYBR Green (Bio-Rad) on a CFX96 Real-Time System (Bio-Rad). Data was analyzed as a fold change utilizing ΔΔCt method normalized to GAPDH expression. Samples were run in triplicate, and three experiments were analyzed. Primer sequences are listed in S1 Table.

### Immunohistochemistry and immunofluorescence

Tissues were embedded, sectioned and stained for hematoxylin and eosin (H&E) by Tufts Histology Core. Necrotic areas were identified and quantified using ImageJ software (NIH). For immunofluorescence (IF), frozen sections were fixed in methanol and treated with 0.1% Triton X-100 (Sigma). Samples were incubated overnight at 4°C with the following primary antibodies diluted with 1.5% goat serum and 1% BSA/PBS: rat monoclonal anti-mouse CD31 (1:200; BD Biosciences; cat. no. 55027), rabbit polyclonal anti-human Ki67 (1:200; Abcam; cat. no. ab15580) or rat monoclonal anti-mouse F4/80 (1:200; eBioscience; cat. no. 17-4801-80). Sections were incubated with goat anti-mouse or anti-rabbit Alexa fluor 546 secondary antibodies (1:250; Invitrogen; anti-mouse A11003; anti-rabbit A11010) for 30 minutes at room temperature. Sections were mounted with Vectashield mounting media with DAPI (Vector Laboratories) and coverslipped. Images were captured using a Nikon Eclipse 80t microscope with SPOT imaging software (Diagnostic Instruments, Inc.).

### Conditioned media

Conditioned media (CM) was collected from control (shCtrl) and shCoR MDA-MB-231 cell lines incubated in basal serum free media (Lonza) (for HUVEC assays) or serum free DMEM (for HL-60 assays) for 18 hours and filtered with a 0.22μm syringe filter prior to use. Adherent HL-60 macrophages were washed with PBS and treated with CM isolated from shCtrl or shCoREST1 MBA-MB-231 cells for 18 hours. Treated HL-60 cells were washed twice with PBS and fed with basal serum free media overnight. HL-60 CM was collected, filter sterilized, and used for assays with HUVECs.

### HUVEC assays

HUVECs in all assays were treated with CM from shCtrl and shCoR MDA-MB-231 cells or CM from HL-60 macrophages activated with CM from shCtrl or shCoR cells. For tube forming assays, 50μl of Matrigel was plated in a 96-well plate and allowed to gel for 30 min at 37°C. 100,000 HUVECs were plated in CM in each well, and tubes formed for 5 hours. Each well was imaged and tubes were quantified using ImageJ. For wound healing assays, 300,000 HUVEC were plated and serum starved overnight. Confluent cells were scratched with a 200μl pipet tip, treated with CM and imaged at 0 hour and 6 hour time points. Wound closure was calculated using ImageJ software and represented as % wound healing. For cell proliferation assays, 50,000 HUVECs/well were plated on a 24 well plate for 6 hr then treated with CM. HUVECs were allowed to proliferate for 72 hr and then counted using the Bio-Rad TC10 Cell Counter. Each condition was plated with 3 replicates in 3 independent experiments for all experiments.

### Macrophage migration assays

For migration assays, 3x10^5^ adherent HL-60 cells were seeded onto 8μm pore size inserts in serum-free DMEM and the inserts were placed in wells containing CM from shCtrl or shCoREST cell lines or CM from shCtrl cells supplemented with 30μg/mL of CCL2 neutralizing antibody (R&D Systems) or 330nM RS504393 (Tocris) for 3 hours. Migrating cells were fixed and stained with crystal violet. Each condition was plated in triplicate, and three experiments were averaged.

### MDA-MB-231 cell proliferation

50,000 shCtrl and shCoREST cells were plated on 12-well plates in 4 replicates. Cells were trypsinized and counted using the Bio-Rad TC10 Cell Counter at 2, 4, and 6 days. Three independent experiments were conducted.

### Cytokine array

shCtrl and shCoREST MDA-MB-231 were plated in equal numbers on 10 cm plates in serum free media and conditioned media collected over 24–48 hr. Supernatants were centrifuged to remove particulates and snap frozen. 1 ml of conditioned media was used to probe a human angiogenesis antibody array (R&D Systems cat. no. ARY007), according to the manufacturer’s instructions.

### Luciferase assay

shCtrl and shCoR #1 MDA-MB-231 cells were plated at a density of 50,000 cell/well on 24-well plates 24 hr prior to transfection. Cells were transfected using Fugene 6HD (Promega) with 400ng of either pMCP-luc (CCL2 promoter in pGL3-basic), which was a gift from Alexander Dent (Addgene plasmid #40324) [34] or VEGF-luc (VEGF promoter in pGL2-basic), which was a gift from Patricia D’Amore (Addgene plasmid #29667) [35] and 50ng of pRL-CMV-Renilla (Promega). After 48 hr, cell lysates were harvested and assayed with the Dual-Glo luciferase assay (Promega) according to the manufacturer’s instructions on a Glomax Multi+ plate reader (Promega). All experiments were conducted in triplicate with 3 biological replicates.

### Statistical analyses

Results from qPCR studies were expressed as mean±s.d. Statistical tests included unpaired two-tailed Student’s t-test or the non-parametric Mann-Whitney test for *in vivo* assays (for 2 groups) and one-way repeated measures ANOVA, followed by multiple comparisons (for more than 2 groups). P values of 0.05 or less were considered to denote significance. Statistical analyses were performed using Graph Pad Prism (Graph Pad Software).

## Results

### Knockdown of CoREST1 inhibits tumorigenesis in a mouse xenograft model of breast cancer

**Fig 1:**
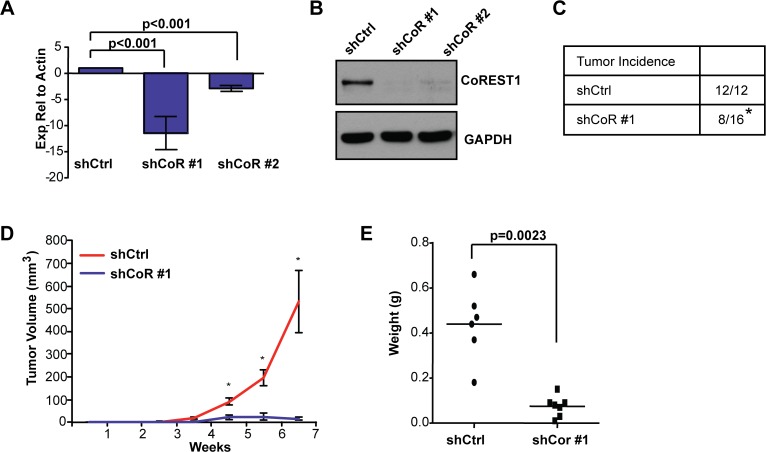
Knockdown of CoREST1 reduced MDA-MB-231 tumor formation. MDA-MB-231 cells were transduced with lentivirus encoding a control (shCtrl) or either of 2 shRNA constructs targeting CoREST1 (shCoR). **(A)** CoREST1 expression in MDA-MB-231 cell lines was quantified using RT-qPCR relative to β-actin expression. Differences were determined using Student’s t-test (n = 6 experiments; mean±s.e.m.). **(B)** Representative image of CoREST1 expression measured by immunoblotting (n>3 experiments). **(C)** NOD/SCID females were injected with shCoR #1 or shCtrl cells into the fourth mammary glands. Masses greater than 3 mm in diameter were defined as tumors (*p<0.001, Fischer’s exact test). **(D)** Tumor growth curve from mice injected with shCoR #1 cells compared to shCtrl controls (*p<0.005, Mann-Whitney test). **(E)** At end stage, tumor weights were measured from mice injected with either shCtrl or shCoR #1 cells. Differences were determined by Mann-Whitney test.

Although CoREST1 has been identified in complexes with factors that promote tumor progression, such as LSD1, ZNF217, ZNF198, HDAC1/2 and SIRT1 [20,36–38], the role of CoREST1 in tumorigenesis is unclear. To investigate the function of CoREST1 in basal-type breast cancer, we utilized two shRNA constructs to stably knockdown CoREST1 in MDA-MB-231 cells. Both constructs significantly reduced CoREST1 transcript and protein levels compared to cells transduced with a control shRNA (shCtrl), although shCoR #1 was more effective at knocking down CoREST1 (Fig. 1A, B). In order to assess how reduced CoREST1 levels affected tumor formation, we injected control or shCoREST1 cells into the mammary fat pads of NOD/SCID mice. Notably, the tumor incidence for mice injected with shCoREST1 cells was reduced to only 50%, in contrast to 100% for mice injected with shCtrl cells (p<0.001; Fig. 1C). In addition, compared to controls, tumors that formed from shCoREST1 cells were significantly smaller in both volume and end stage weight (Fig. 1D, E). These findings demonstrate that depletion of CoREST1 in MDA-MB-231 cells impaired tumor formation and growth *in vivo*.

**Fig 2:**
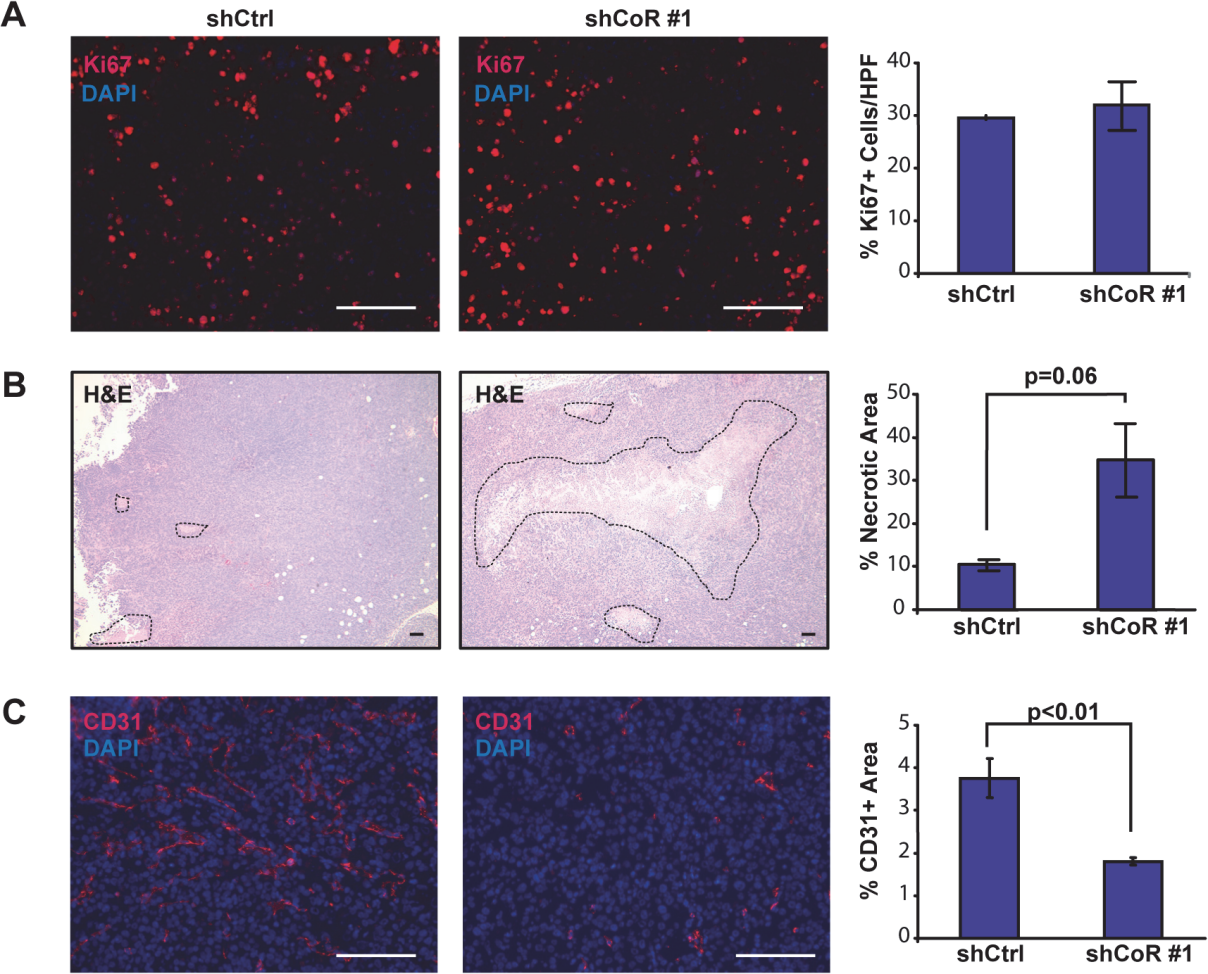
Decreased CoREST1 expression reduced tumor angiogenesis. **(A)** Ki67 expression, a marker of proliferation, was detected in control (shCtrl) and shCoREST1 (shCoR #1) tumors. Percent Ki67 positive nuclei per high power field (HPF) were quantified in three images from each tumor using ImageJ. In this image, Ki67 immunostaining is red and DAPI (to detect nuclei) is blue. **(B)** Necrosis was quantified on hematoxylin and eosin (H&E) stained slides in tumors that formed from either shCoR or shCtrl cells. Differences were determined using Student’s t-test (n = 6 tumors/group). **(C)** CD31 expression was detected using immunofluorescence in shCoR or shCtrl tumors. CD31 expression (red) was quantified using five high power fields of DAPI positive nuclei (blue) from each tumor. Differences were determined using Student’s t-test (n = 3 tumors/group). Scale bar = 100μm.

Pharmacological inhibition or knockdown of the CoREST1 associated factor LSD1 has been shown to inhibit proliferation in several breast cancer cell lines, including MDA-MB-231 cells [16,17,39]. CoREST1 has been suggested to regulate LSD1 levels and stability [40] and, consistent with this, we observed reduced LSD1 levels in shCoREST1 cells compared to controls (S1 Fig.). Thus, we considered the possibility that knockdown of CoREST1 expression may lead to diminished cellular proliferation in MDA-MB-231 cells. However, *in vitro*, we observed no significant differences in cellular proliferation or morphology in shCoREST1 cells compared with control cells (S1 Fig.). Further, tumors that formed from shCoREST1 cells demonstrated similar levels of the proliferation marker Ki67 compared with tumors that formed from control cells (Fig. 2A). Taken together, these results suggest that the striking inhibition of tumor formation *in vivo* observed upon CoREST1 knockdown was not due to reduced cellular proliferation.

Although shCoREST1 tumors were histologically similar to tumors that formed from control cells, shCoREST1 tumors had increased areas of focal necrosis compared with control tumors (Fig. 2B). The presence of these large necrotic areas suggested that reduced CoREST1 expression in the tumor cells may have led to diminished angiogenesis within the tumor parenchyma. Immunostaining for CD31, an endothelial cell marker, revealed that vascular density was significantly reduced in CoREST1 depleted tumors (p<0.01; Fig. 2C). These observations suggest that CoREST1 expression may promote tumor growth by enhancing angiogenesis in the tumor microenvironment.

### CoREST1 regulates the tumor cell secretome

**Fig 3:**
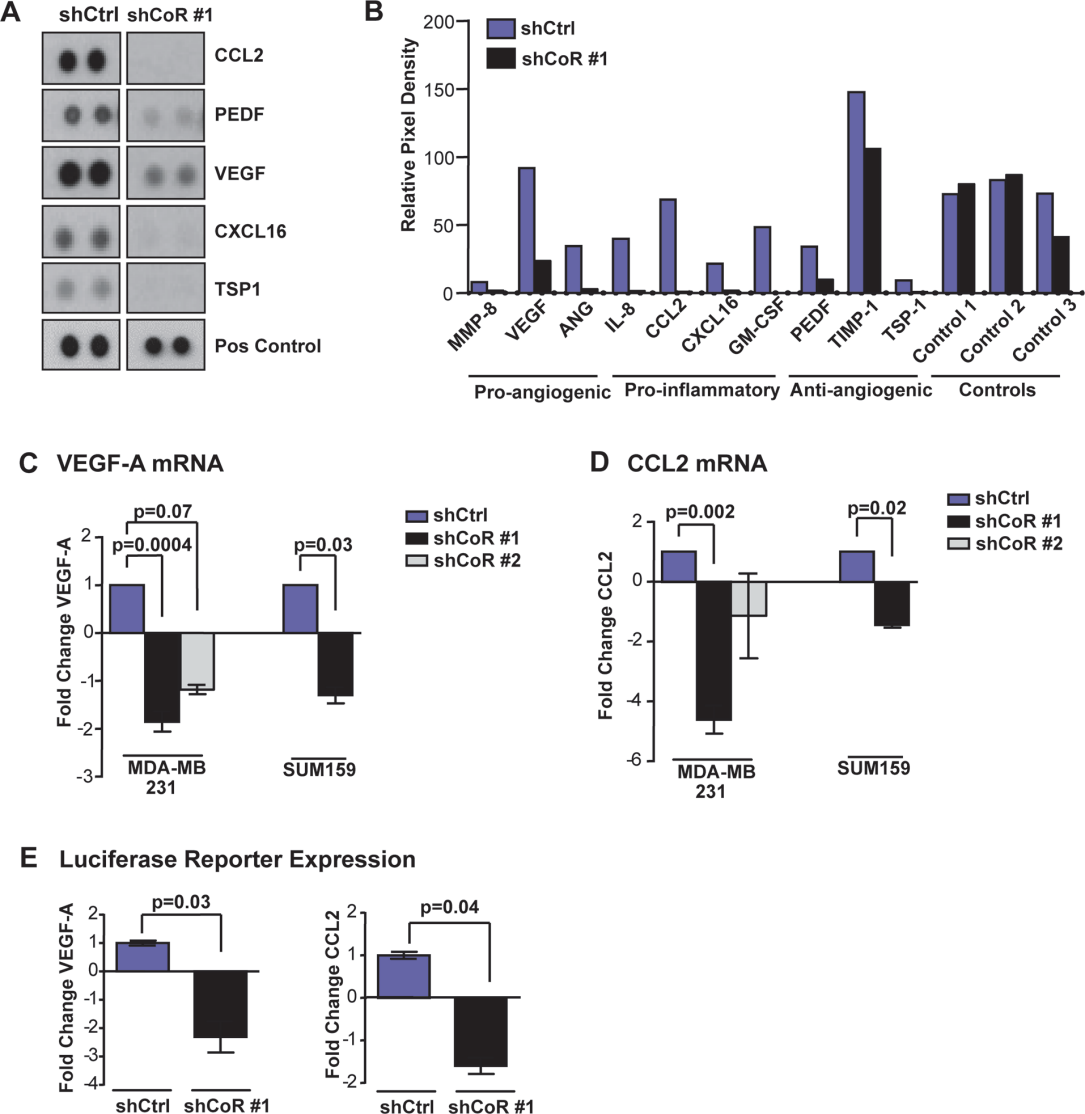
Depletion of CoREST1 altered the tumor cell secretome. **(A)** Conditioned media from shcontrol (shCtrl) and shCoREST1 (shCoR #1) MDA-MB-231 cells was incubated with a human angiogenesis antibody array as described in Materials and Methods. Immunoblot images from this screen, performed one time, are shown. **(B)** Quantification of the relative pixel density on the array for the indicated pro-angiogenic, pro-inflammatory and anti-angiogenic factors secreted by shCtrl and shCoR cell lines (n = 1 experiment). **(C)** VEGF-A mRNA expression was measured in shCoR cells compared to shCtrl cells in MDA-MB-231 and SUM159 cell lines. **(D)** CCL2 mRNA expression was measured in the indicated cell lines. Expression levels were detected by RT-qPCR and represented as fold change compared to shCtrl cells. Differences were determined by Student’s t-test (mean±s.d.; n = 3 experiments). **(E)** Luciferase activity from shCtrl or shCoR #1 MDA-MB-231 cells transfected with VEGF-luc or pMCP-luc and pRL-CMV-Renilla. Luciferase expression was normalized to Renilla, then expressed as fold change compared to shCtrl cells. Differences were determined by Student’s t-test (mean±s.d.; n = 3 experiments).

We hypothesized that CoREST1 might modulate the tumor microenvironment through the regulated expression of factors secreted by the tumor cell. Therefore, we carried out a screen to compare the secretomes of shCoREST1 and control MDA-MB-231 cells using a human angiogenesis antibody array that allowed for simultaneous evaluation of 55 secreted factors (S2 Table). CoREST1 knockdown resulted in striking changes in the tumor cell conditioned media including notable decreases in levels of secreted pro-angiogenic factor vascular endothelial growth factor A (VEGF-A), pro-inflammatory factors CCL2/MCP-1 and CXCL16, as well as anti-angiogenic factor thrombospondin 1 (TSP1) compared to conditioned media from control cells (Fig. 3A, B). Thus, knockdown of CoREST1 leads to striking changes in of the levels of both pro- and anti- angiogenic and inflammatory factors secreted by these breast cancer cells.

Since CoREST1 is known to regulate chromatin structure and gene expression, we investigated whether some of the observed changes in levels of secreted angiogenic and inflammatory factors occurred at the mRNA level. RT-qPCR analyses confirmed that mRNA expression of several factors was altered in shCoREST1 cells (S2 Fig.). In particular, we observed that VEGF-A and CCL2 mRNA levels were reduced in shCoREST1 MDA-MB-231 cells (Fig. 3C, D). VEGF-A and CCL2 expression were also significantly reduced in response to CoREST1 knockdown in another basal-type breast cancer cell line, SUM159 (Fig. 3C, D). Similar to these effects on endogenous mRNAs, we also observed that CoREST1 knockdown reduced expression from luciferase reporters bearing the 5’ promoter regions of either VEGFA or CCL2 (Fig. 3E). Together, these results show that CoREST1 acts in at least some basal tumor cells to promote the expression of multiple factors expected to influence the tumor microenvironment.

### CoREST1 in tumor cells promotes non-cell autonomous effects on endothelial cells

**Fig 4:**
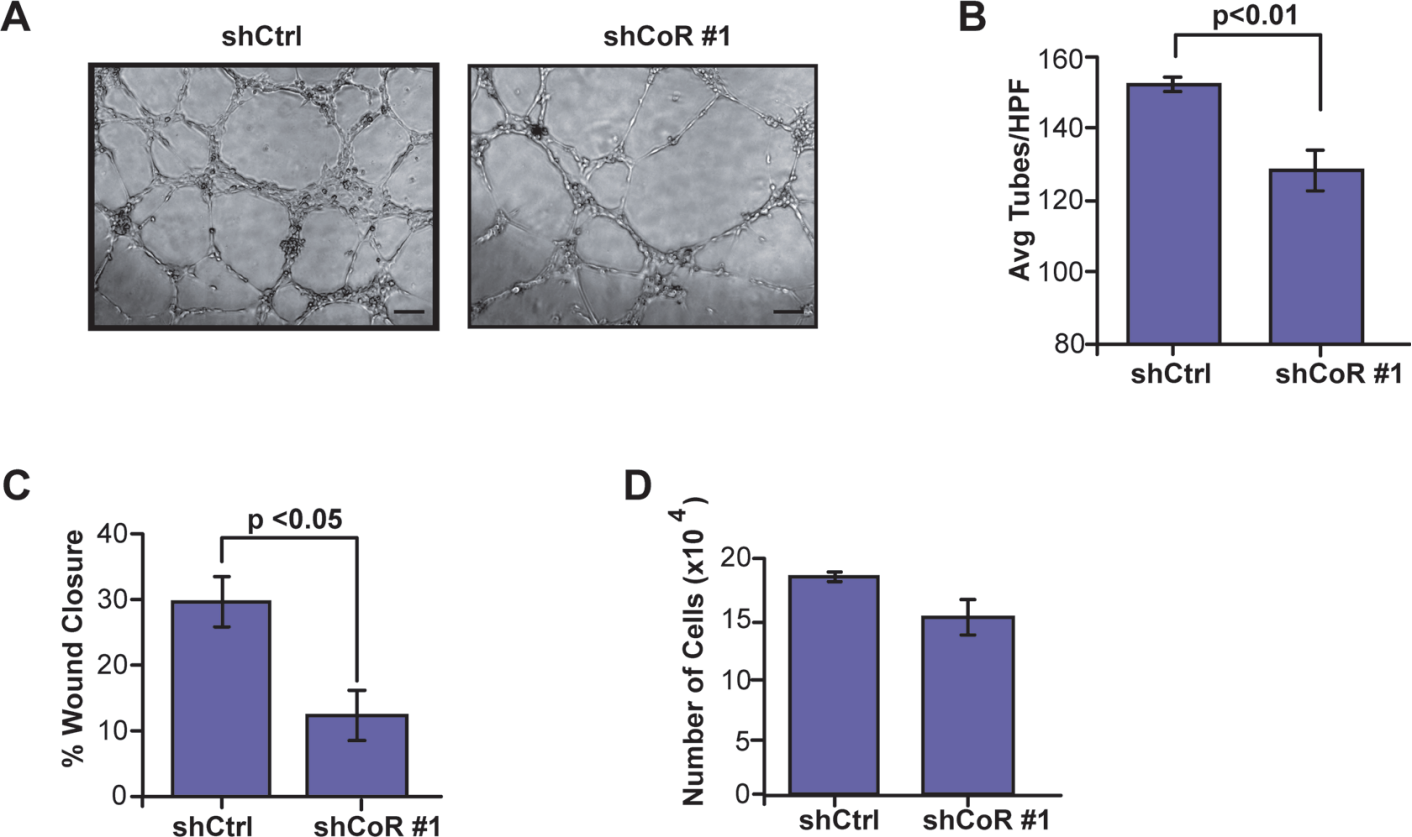
Knockdown of CoREST1 decreased tumor cell-mediated stimulation of endothelial cells *in vitro*. **(A)** HUVECs were grown in conditioned media (CM) from shCoREST1 (shCoR) or control (shCtrl) cells on Matrigel for 6 hr to assess changes in tube forming ability. **(B)** Quantification of tube formation of HUVEC treated with shCtrl or shCoR CM. Tubes from 5 high power fields (HPF) were averaged for each condition tested (n = 3 experiments). **(C)** HUVEC cells were exposed to CM from shCtrl cells or shCoR cells and wound closure was measured 6 hr after scratching confluent cells as described in Materials and Methods. Data is expressed as % of wound closure as determined from an average of 10 replicates per condition (n = 3 experiments). **(D)** Proliferation of HUVEC cells after exposure to shCtrl or shCoR CM was determined by counting cells after 72 hours (n = 3 experiments). Differences were determined by Student’s t-test (mean±s.d.). Scale bar = 100μm.

Given the pro-angiogenic role of many of the factors with reduced abundance in the secretome of shCoREST1 cells compared with control cells, we hypothesized that CoREST1 regulates signaling to endothelial cells. We therefore investigated the effects of conditioned media from MD-MBA-231 on human umbilical vein endothelial cells (HUVECs). We exposed HUVECS to conditioned media from control and shCoREST1 MDA-MB-231 cells and measured endothelial tube formation. Conditioned media from shCoREST1 cells significantly reduced tube formation compared with conditioned media from control cells (p<0.01; Fig. 4A, B). Further, conditioned media from shCoREST1 cells significantly reduced HUVEC migration in a wound healing assay (p<0.05; Fig. 4C). No significant differences were detected in the proliferation rate of HUVECs following treatment with conditioned media from shCoREST1 or control cells (Fig. 4D). These results suggest that the altered secretome of shCoREST1 breast cancer cells limited endothelial migration and differentiation to form new blood vessels. Further, these *in vitro* data suggest that CoREST1 alters angiogenesis within the tumor microenvironment through modulation of the tumor cell secretome.

### CoREST1 modulates angiogenesis through macrophage recruitment

**Fig 5:**
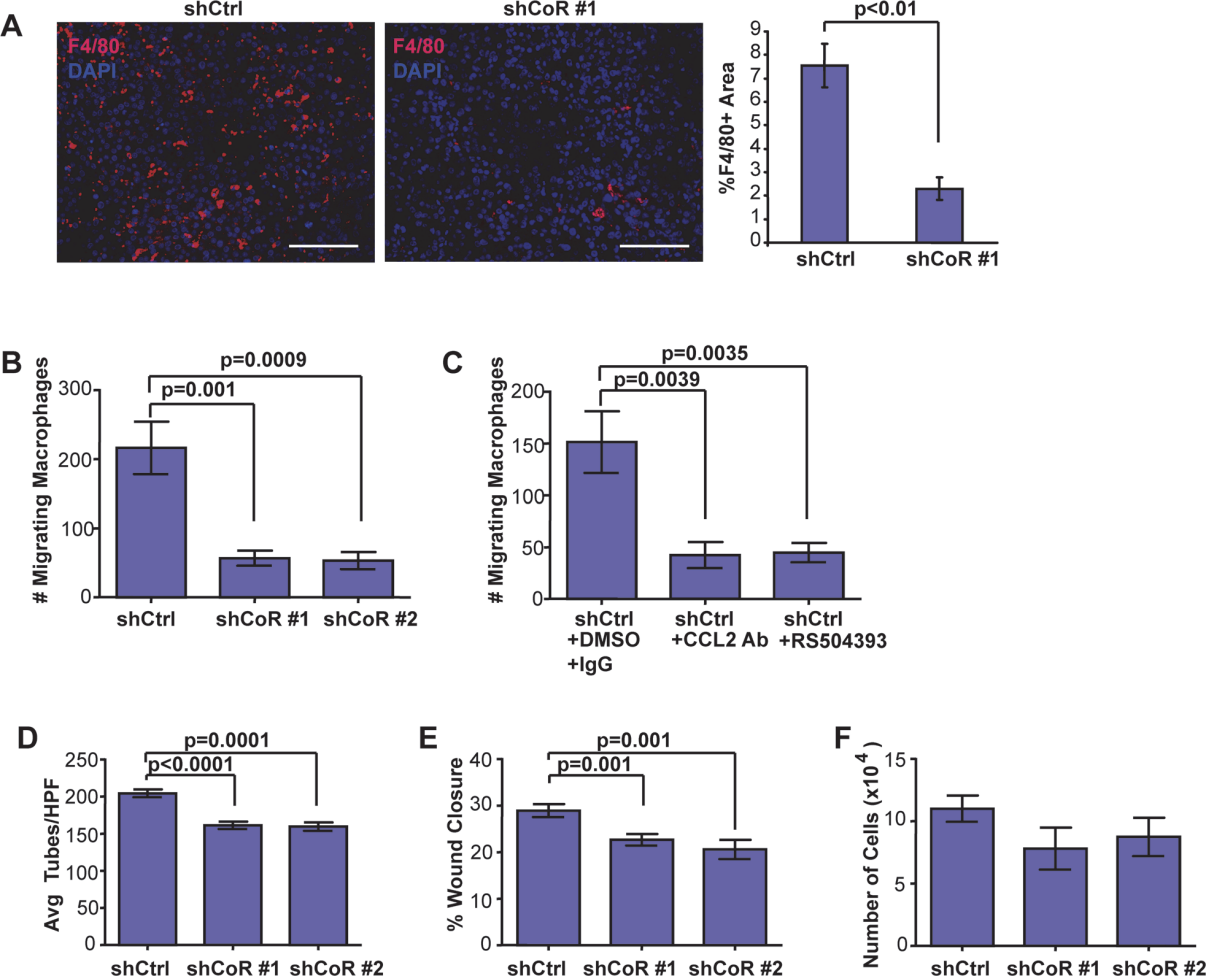
Knockdown of CoREST1 decreased tumor cell-mediated macrophage migration and activation. **(A)** F4/80 immunostaining, a marker of macrophage infiltration, was performed in tumors that grew from shCtrl and shCoREST (shCoR) cells. F4/80 expression (red) was quantified using ImageJ using five high power fields of DAPI positive nuclei (blue) from each tumor. Differences were determined using Student’s t-test (n = 3 tumors/group). **(B)** Migration of HL-60 macrophages was measured in response to conditioned media (CM) from shCoR cells compared to shCtrl cells. HL-60 cells were differentiated into macrophages as described in Materials and Methods. Transwell migration of macrophages was quantified after 4 hr, and differences were determined by ANOVA analysis (n = 3 experiments in triplicate). **(C)** Migration of HL-60 macrophages was examined in response to CM from shCtrl cells supplemented with vehicle, a blocking antibody to CCL2, or RS504393, an inhibitor for the CCR2 receptor. Transwell migration of macrophages was quantified after 4 hr, and differences were determined by ANOVA analysis (n = 3 experiments in triplicate). **(D)** HUVEC tube formation was examined in response to CM collected from HL-60 macrophages activated with CM from either shCoR or shCtrl cells. Tubes from 3 high power fields (HPF) were averaged for each condition tested, and differences were determined by ANOVA analysis (n = 3 experiments). **(E)** HUVEC cell migration was measured following treatment with CM from macrophages activated with CM isolated from either shCoR or shCtrl cells. Wound closure was measured using ImageJ software 6 hr after scratching confluent cells as described in Materials and Methods. Data is expressed as % of wound closure as determined from an average of 10 replicates per condition (n = 3 experiments). **(F)** Proliferation of HUVEC cells was not altered in response to treatment with CM from macrophages activated with either shCtrl or shCoR CM. HUVEC were counted after 72 hr (n = 3 experiments). Differences were determined by ANOVA analysis (mean±s.d.). Scale bar = 100μm.

Macrophages play a key role in tumor angiogenesis (for review, [7,41]). Although CCL2 has been shown to have direct effects on endothelial cells and angiogenesis in some models [42–44], CCL2 was first characterized as a potent chemoattractant for macrophages (for review, [45]). We therefore hypothesized that decreased secretion of CCL2, and possibly other factors, by shCoREST1 cells could contribute to reduced angiogenesis through modulation of macrophages in the shCoREST1 tumor microenvironment. We stained shCoREST1 and control tumors for F4/80, a macrophage marker, and quantified expression. Compared with control tumors, shCoREST1 tumors demonstrated significantly decreased macrophage recruitment (p<0.01; Fig. 5A). *In vitro*, the migration of HL-60-derived macrophages was reduced in response to conditioned media from shCoREST1 cells compared with conditioned media from control cells in transwell assays (Fig. 5B). Consistent with a key role for CCL2 in this process, macrophage migration was significantly reduced in the presence of a blocking antibody for CCL2 (p = 0.0039) as well as upon addition of RS504393, a small molecule inhibitor for the receptor of CCL2 (p = 0.0035; Fig. 5C). These data suggest that one way that CoREST1 modulates the tumor microenvironment is through the recruitment of macrophages via regulation of CCL2 expression.

Since tumor activated macrophages (TAMs) secrete factors that promote angiogenesis, we also compared HL-60-derived macrophages activated with conditioned media from either shCoREST1 or control breast cancer cells. We collected conditioned media from the activated macrophages and tested the ability of their secreted factors to promote the growth and migration of HUVEC cells. Compared with macrophages treated with control conditioned media, macrophages primed with conditioned media from shCoREST1 cells had significantly reduced ability to promote HUVEC tube-formation as well as migration in a wound healing assay (Fig. 5D, E). Similar to treatment of HUVEC with conditioned media from shCoREST1 tumor cells, shCoREST1 activated macrophage conditioned media did not significantly alter HUVEC proliferation (Fig. 5F). Together, these data suggest that CoREST1 acts in breast tumor cells to alter the tumor secretome, thereby promoting tumor vascularity through both tumor cell-mediated angiogenesis as well as through the recruitment and activation of pro-angiogenic macrophages.

## Discussion

Developing tumors require interactions with the surrounding microenvironment for progression to malignancy. Our findings reveal that the transcriptional regulator CoREST1 promotes tumorigenesis by enhancing angiogenesis. We found that CoREST1 regulates the expression of tumor cell secreted factors to promote angiogenesis through direct effects on endothelial cells as well as indirect effects via the recruitment and activation of tumor associated macrophages (TAMs). Knockdown of CoREST1 in MDA-MB-231 cells decreased the incidence and reduced the size of tumors in an *in vivo* xenograft model. Histological examination of the shCoREST1 tumors revealed significantly reduced recruitment of both endothelial cells and macrophages. These changes in the tumor microenvironment correlated with reduced expression of pro-angiogenic and pro-inflammatory factors in CoREST1 knockdown cells. Our study implicates CoREST1 in both angiogenesis and the recruitment and activation of TAMs. Our cell culture and *in vivo* data significantly add to the understanding of CoREST1 in tumorigenesis beyond its described biochemical functions.

Angiogenesis is essential for the growth of solid tumors. VEGF-A is a well-studied pro-angiogenic factor, and high levels of VEGF-A in breast cancers have been correlated with poor prognosis [46–48]. We found that VEGF-A mRNA and protein were reduced by knockdown of shCoREST1 in MDA-MB-231 basal-type breast cancer cells. Consistent with reduced VEGF-A expression, we found that conditioned media from shCoREST1 cells had a reduced ability to promote HUVEC migration and organization into endothelial tubes compared with control cells. CoREST1 knockdown altered levels of many secreted factors in addition to VEGF-A, for example anti-angiogenic factors like PEDF were also downregulated. Thus, the overall change in the levels and balance of pro- and anti-angiogenic factors altered by CoREST1 knockdown likely contributes to CoREST1-dependent regulation of the tumor microenvironment.

Cancer cells secrete a number of cytokines and chemokines that attract endothelial cells and inflammatory cells necessary to support growth of the tumor. Macrophages, in turn, secrete cytokines, which can promote the expansion of aggressive cancer stem-like cell populations within tumors [49–52] as well as angiogenesis [7,41]. Knockdown of CoREST1 in MDA-MB-231 breast cancer cells resulted in downregulation of several pro-inflammatory factors at both the RNA and protein level, including CCL2, one of the key chemokines that promotes infiltration of macrophages and monocytes into the tumor microenvironment [45]. These changes in cytokine levels were associated with reduced ability of conditioned media from CoREST1 knockdown cells to promote macrophage migration and activation of pro-angiogenic properties *in vitro*. Reduced CCL2 expression may also contribute to the decreased recruitment of F4/80^+^ macrophages to shCoREST1 cell-derived tumors observed *in vivo*. Thus, our data suggest that CoREST1 regulates both direct and indirect mechanisms of endothelial cell recruitment to tumors.

Our data support the view that the CoREST1-mediated changes in the tumor cell secretome occur at the transcriptional level. Further, despite the well described function of CoREST1 as a corepressor, our findings suggest that CoREST1 functions as an activator of angiogenic and inflammatory genes such as VEGF-A and CCL2. CoREST1 may activate gene expression indirectly; for example, CoREST1 may repress expression of an inhibitor, such as a miRNA, and upon CoREST knockdown, levels of the inihibitor increase which in turn leads to reduced levels of VEGF-A and CCL2 expression. It also possible that knockdown of CoREST1 indirectly impacts gene expression by altering the relative abundance of LSD1 complexes with the other CoREST family members, since CoREST2/LSD1 and CoREST3/LSD1 complexes have some distinct activities [53–56]. Direct activation models cannot be ruled out, however, as both LSD1 and CoREST1 have been reported to activate transcription in some contexts; activation has been suggested to occur through LSD1-mediated demethylation of substrates other than mono- and di-methylated H3K4 [30,31,57].

The role of CoREST1 in tumor/stroma interactions likely requires the known biochemical function of CoREST1 to promote recruitment and activity of associated histone modifying enzymes including LSD1 and/or HDAC1/2. In cancer cells, the HDAC inhibitor, TSA, reduced both protein and mRNA levels of VEGF-A [58]. CoREST1 is intimately associated with LSD1 and is required for demethylase activity on nucleosomes [10,28]. We observed reduced LSD1 levels in CoREST1 knockdown cells (S1 Fig.), further supporting the model that reduced LSD1 activity may contribute to the observed CoREST1 knockdown phenotypes. Similar to our findings with CoREST1 knockdown, depletion of LSD1 in prostate cancer cells was reported to reduce VEGF-A mRNA levels [59]. Curiously, these results were not recapitulated using an inhibitor for LSD1, raising the possibility that the enzymatic activity of LSD1 may not be required for the regulation of VEGF-A expression [59]. Although knockdown and/or inhibition of LSD1 in cancer cells resulted in decreased proliferation [15–17,39,60] and tumor growth [12,61], our data show that knockdown of CoREST1 in MDA-MB-231 breast cancer cells did not reduce proliferation. We consider it likely that CoREST1 knockdown may have gene-specific effects on LSD1 activity, quite distinct from global inhibition or loss of LSD1. Consistent with this idea, as noted above, LSD1 can function in complex with other CoREST homologs and also as part of a distinct LSD1/NuRD complex [53,54,62]. It is also possible that CoREST1 has some LSD1-independent functions, as the Drosophila CoREST homolog has been reported to function in complexes independent of LSD1 [63]. Thus, additional studies, including examining histone methylation and acetylation at specific promoters, are required to determine whether CoREST1 functions together with or independently of LSD1 and/or HDAC1/2 in promoting tumor angiogenesis.

## Conclusions

Taken together, our data support a role for CoREST1 in regulating expression of tumor cell secreted factors to promote inflammation and angiogenesis. Although additional studies are needed to determine the significance of this mechanism in other tumor types and human populations, nonetheless, these data implicate a new player in epigenetic control of tumor/stroma interactions. Further understanding of the mechanisms of how tumor cells regulate angiogenesis in their microenvironment will provide new insights into tumor growth and progression and could lead to novel therapies.

## Supporting Information

S1 Fig Depletion of CoREST1 in MDA-MB-231 cells does not induce changes in proliferation or cell morphology *in vitro*. **(A)** Immunoblotting of LSD1 and CoREST1 in lysates from MDA-MB-231 breast cancer cells stably transfected with control (shCtrl) or either of 2 shRNA constructs targeting CoREST1 (shCoR). **(B)** Proliferation rates of control (shCtrl) and shCoREST1 (shCoR) MDA-MB-231 cell lines were not significantly different. Cells were plated and quantified at the indicated times as described in Materials and Methods. **(C)** No significant differences were observed in cell morphology in MDA-MB-231 cells with and without CoREST1 depletion. Scale bar = 100μm.
(TIF)

S2 Fig Depletion of CoREST1 alters expression of genes in MDA-MB-231 cells. Expression levels of the indicated genes were quantified from control (shCtrl) or CoREST1 depleted (shCoR #1 and shCoR #2) MDA-MB-231 cells using RT-qPCR. Values represented as a fold change compared to shCtrl cells.
(TIF)

S1 Table Primers used for RT-qPCR analysis. (DOCX)

S2 Table Factors quantified using Human Angiogenesis Array. Conditioned media from shcontrol (shCtrl) and shCoREST1 (shCoR #1) MDA-MB-231 cells was incubated with a human angiogenesis antibody array as described in Materials and Methods. Quantification of the relative pixel density for all of the factors present on the array (n = 1 experiment).
(DOCX)

## References

[1] Hanahan D, Weinberg RA. Hallmarks of cancer: the next generation. Cell. 2011;144: 646–674. 10.1016/j.cell.2011.02.013 21376230

[2] Arendt LM, Rudnick JA, Keller PJ, Kuperwasser C. Stroma in breast development and disease. Semin Cell Dev Biol. 2010;21: 11–18. 10.1016/j.semcdb.2009.10.003 19857593 PMC2823823

[3] Orimo A, Weinberg RA. Stromal fibroblasts in cancer: a novel tumor-promoting cell type. Cell Cycle. 2006;5: 1597–1601. 16880743 10.4161/cc.5.15.3112

[4] Dvorak HF. Tumors: wounds that do not heal. Similarities between tumor stroma generation and wound healing. N Engl J Med. 1986;315: 1650–1659. 3537791 10.1056/NEJM198612253152606

[5] Mao Y, Keller ET, Garfield DH, Shen K, Wang J. Stromal cells in tumor microenvironment and breast cancer. Cancer Metastasis Rev. 2013;32: 303–315. 10.1007/s10555-012-9415-3 23114846 PMC4432936

[6] Porta C, Subhra KB, Larghi P, Rubino L, Mancino A, Sica A. Tumor promotion by tumor-associated macrophages. Adv Exp Med Biol. 2007;604: 67–86. 17695721 10.1007/978-0-387-69116-9_5

[7] Qian BZ, Pollard JW. Macrophage diversity enhances tumor progression and metastasis. Cell. 2010;141: 39–51. 10.1016/j.cell.2010.03.014 20371344 PMC4994190

[8] Laugesen A, Helin K. Chromatin repressive complexes in stem cells, development, and cancer. Cell Stem Cell. 2014;14: 735–751. 10.1016/j.stem.2014.05.006 24905164

[9] Witt O, Deubzer HE, Milde T, Oehme I. HDAC family: What are the cancer relevant targets? Cancer Lett. 2009;277: 8–21. 10.1016/j.canlet.2008.08.016 18824292

[10] Shi YJ, Matson C, Lan F, Iwase S, Baba T, Shi Y. Regulation of LSD1 histone demethylase activity by its associated factors. Mol Cell. 2005;19: 857–864. 16140033 10.1016/j.molcel.2005.08.027

[11] Shi Y, Lan F, Matson C, Mulligan P, Whetstine JR, Cole PA, et al Histone demethylation mediated by the nuclear amine oxidase homolog LSD1. Cell. 2004;119: 941–953. 15620353 10.1016/j.cell.2004.12.012

[12] Schulte JH, Lim S, Schramm A, Friedrichs N, Koster J, Versteeg R, et al Lysine-specific demethylase 1 is strongly expressed in poorly differentiated neuroblastoma: implications for therapy. Cancer Res. 2009;69: 2065–2071. 10.1158/0008-5472.CAN-08-1735 19223552

[13] Serce N, Gnatzy A, Steiner S, Lorenzen H, Kirfel J, Buettner R. Elevated expression of LSD1 (Lysine-specific demethylase 1) during tumour progression from pre-invasive to invasive ductal carcinoma of the breast. BMC Clin Pathol. 2012;12: 13 10.1186/1472-6890-12-13 22920283 PMC3511290

[14] Lv T, Yuan D, Miao X, Lv Y, Zhan P, Shen X, et al Over-expression of LSD1 promotes proliferation, migration and invasion in non-small cell lung cancer. PLoS One. 2009;7: e35065. 10.1371/journal.pone.0035065 PMC3320866 22493729

[15] Huang Y, Vasilatos SN, Boric L, Shaw PG, Davidson NE. Inhibitors of histone demethylation and histone deacetylation cooperate in regulating gene expression and inhibiting growth in human breast cancer cells. Breast Cancer Res Treat. 2012;131: 777–789. 10.1007/s10549-011-1480-8 21452019 PMC3624096

[16] Lim S, Janzer A, Becker A, Zimmer A, Schule R, Buettner R, et al Lysine-specific demethylase 1 (LSD1) is highly expressed in ER-negative breast cancers and a biomarker predicting aggressive biology. Carcinogenesis. 2010;31: 512–520. 10.1093/carcin/bgp324 20042638

[17] Zhu Q, Huang Y, Marton LJ, Woster PM, Davidson NE, Casero RA Jr. Polyamine analogs modulate gene expression by inhibiting lysine-specific demethylase 1 (LSD1) and altering chromatin structure in human breast cancer cells. Amino Acids. 2012;42: 887–898. 10.1007/s00726-011-1004-1 21805138 PMC3240695

[18] Ferrari-Amorotti G, Chiodoni C, Shen F, Cattelani S, Soliera AR, Manzotti G, et al Suppression of invasion and metastasis of triple-negative breast cancer lines by pharmacological or genetic inhibition of slug activity. Neoplasia. 2014;16: 1047–1058. 10.1016/j.neo.2014.10.006 25499218 PMC4557365

[19] Humphrey GW, Wang Y, Russanova VR, Hirai T, Qin J, Nakatani Y, et al Stable histone deacetylase complexes distinguished by the presence of SANT domain proteins CoREST/kiaa0071 and Mta-L1. J Biol Chem. 2001;276: 6817–6824. 11102443 10.1074/jbc.M007372200

[20] You A, Tong JK, Grozinger CM, Schreiber SL. CoREST is an integral component of the CoREST- human histone deacetylase complex. Proc Natl Acad Sci U S A. 2001;98: 1454–1458. 11171972 10.1073/pnas.98.4.1454 PMC29278

[21] Andres ME, Burger C, Peral-Rubio MJ, Battaglioli E, Anderson ME, Grimes J, et al CoREST: a functional corepressor required for regulation of neural-specific gene expression. Proc Natl Acad Sci U S A. 1999;96: 9873–9878. 10449787 10.1073/pnas.96.17.9873 PMC22303

[22] Ballas N, Battaglioli E, Atouf F, Andres ME, Chenoweth J, Anderson ME, et al Regulation of neuronal traits by a novel transcriptional complex. Neuron. 2001;31: 353–365. 11516394 10.1016/s0896-6273(01)00371-3

[23] Hakimi MA, Bochar DA, Chenoweth J, Lane WS, Mandel G, Shiekhattar R. A core-BRAF35 complex containing histone deacetylase mediates repression of neuronal-specific genes. Proc Natl Acad Sci U S A. 2001;99: 7420–7425. 10.1073/pnas.112008599 PMC124246 12032298

[24] Saleque S, Kim J, Rooke HM, Orkin SH. Epigenetic regulation of hematopoietic differentiation by Gfi-1 and Gfi-1b is mediated by the cofactors CoREST and LSD1. Mol Cell. 2007;27: 562–572. 17707228 10.1016/j.molcel.2007.06.039

[25] Lin Y, Wu Y, Li J, Dong C, Ye X, Chi YI, et al The SNAG domain of Snail1 functions as a molecular hook for recruiting lysine-specific demethylase 1. EMBO J. 2010;29: 1803–1816. 10.1038/emboj.2010.63 20389281 PMC2885925

[26] Lin T, Ponn A, Hu X, Law BK, Lu J. Requirement of the histone demethylase LSD1 in Snai1-mediated transcriptional repression during epithelial-mesenchymal transition. Oncogene. 2010;29: 4896–4904. 10.1038/onc.2010.234 20562920 PMC3093107

[27] Tsai MC, Manor O, Wan Y, Mosammaparast N, Wang JK, Lan F, et al Long noncoding RNA as modular scaffold of histone modification complexes. Science. 2010;329: 689–693. 10.1126/science.1192002 20616235 PMC2967777

[28] Lee MG, Wynder C, Cooch N, Shiekhattar R. An essential role for CoREST in nucleosomal histone 3 lysine 4 demethylation. Nature. 2005;437: 432–435. 16079794 10.1038/nature04021

[29] Lee MG, Wynder C, Bochar DA, Hakimi MA, Cooch N, Shiekhattar R. Functional interplay between histone demethylase and deacetylase enzymes. Mol Cell Biol. 2006;26: 6395–6402. 16914725 10.1128/MCB.00723-06 PMC1592851

[30] Metzger E, Wissmann M, Yin N, Muller JM, Schneider R, Peters AH, et al LSD1 demethylates repressive histone marks to promote androgen-receptor-dependent transcription. Nature. 2005;437: 436–439. 16079795 10.1038/nature04020

[31] Cai C, He HH, Gao S, Chen S, Yu Z, Gao Y, et al Lysine-specific demethylase 1 has dual functions as a major regulator of androgen receptor transcriptional activity. Cell Rep. 2014;9: 1618–1627. 10.1016/j.celrep.2014.11.008 25482560 PMC4268354

[32] Arendt LM, McCready J, Keller PJ, Baker DD, Naber SP, Seewaldt V, et al Obesity promotes breast cancer by CCL2-mediated macrophage recruitment and angiogenesis. Cancer Res. 2013;73:6080–6093. 10.1158/0008-5472.CAN-13-0926 23959857 PMC3824388

[33] Keller PJ, Arendt LM, Skibinski A, Logvinenko T, Klebba I, Dong S, et al Defining the cellular precursors to human breast cancer. Proc Natl Acad Sci U S A. 2011;109:2772–2777. 10.1073/pnas.1017626108 21940501 PMC3286919

[34] Toney LM, Cattoretti G, Graf JA, Merghoub T, Pandolfi PP, Dalla-Favera R, et al BCL-6 regulates chemokine gene transcription in macrophages. Nat Immunol. 2011;1: 214–220. 10.1038/79749 10973278

[35] Shima DT, Kuroki M, Deutsch U, Ng YS, Adamis AP, D'Amore PA. The mouse gene for vascular endothelial growth factor. Genomic structure, definition of the transcriptional unit, and characterization of transcriptional and post-transcriptional regulatory sequences. J Biol Chem. 1996;271: 3877–3883. 8632007 10.1074/jbc.271.7.3877

[36] Gocke CB, Yu H. ZNF198 stabilizes the LSD1-CoREST-HDAC1 complex on chromatin through its MYM-type zinc fingers. PLoS One. 2008;3: e3255 10.1371/journal.pone.0003255 18806873 PMC2532748

[37] Mulligan P, Yang F, Di SL, Ji JY, Ouyang J, Nishikawa JL, et al SIRT1-LSD1 corepressor complex regulates Notch target gene expression and development. Mol Cell. 2011;42: 689–699. 10.1016/j.molcel.2011.04.020 21596603 PMC3119599

[38] Thillainadesan G, Isovic M, Loney E, Andrews J, Tini M, Torchia J. Genome analysis identifies the p15ink4b tumor suppressor as a direct target of the ZNF217/CoREST complex. Mol Cell Biol. 2008;28: 6066–6077. 10.1128/MCB.00246-08 18625718 PMC2547005

[39] Pollock JA, Larrea MD, Jasper JS, McDonnell DP, McCafferty DG. Lysine-specific histone demethylase 1 inhibitors control breast cancer proliferation in ERalpha-dependent and -independent manners. ACS Chem Biol. 2012;7: 1221–1231. 10.1021/cb300108c 22533360 PMC3582702

[40] LaMarca HL, Visbal AP, Creighton CJ, Liu H, Zhang Y, Behbod F, et al CCAAT/enhancer binding protein beta regulates stem cell activity and specifies luminal cell fate in the mammary gland. Stem Cells. 2010;28: 535–544. 10.1002/stem.297 20054865 PMC3006225

[41] Guo C, Buranych A, Sarkar D, Fisher PB, Wang XY. The role of tumor-associated macrophages in tumor vascularization. Vasc Cell. 2013;5: 20 10.1186/2045-824X-5-20 24314323 PMC3913793

[42] Salcedo R, Ponce ML, Young HA, Wasserman K, Ward JM, Kleinman HK, et al Human endothelial cells express CCR2 and respond to MCP-1: direct role of MCP-1 in angiogenesis and tumor progression. Blood. 2000;96: 34–40. 10891427

[43] Gordillo GM, Onat D, Stockinger M, Roy S, Atalay M, Beck FM, et al A key angiogenic role of monocyte chemoattractant protein-1 in hemangioendothelioma proliferation. Am J Physiol Cell Physiol. 2004;287: C866–C873. 15163622 10.1152/ajpcell.00238.2003

[44] Stamatovic SM, Keep RF, Mostarica-Stojkovic M, Andjelkovic AV. CCL2 regulates angiogenesis via activation of Ets-1 transcription factor. J Immunol. 2006;177: 2651–2661. 16888027 10.4049/jimmunol.177.4.2651

[45] Deshmane SL, Kremlev S, Amini S, Sawaya BE. Monocyte chemoattractant protein-1 (MCP-1): an overview. J Interferon Cytokine Res. 2009;29: 313–326. 10.1089/jir.2008.0027 19441883 PMC2755091

[46] Gasparini G, Toi M, Gion M, Verderio P, Dittadi R, Hanatani M, et al Prognostic significance of vascular endothelial growth factor protein in node-negative breast carcinoma. J Natl Cancer Inst. 1997;89: 139–147. 8998183 10.1093/jnci/89.2.139

[47] Foekens JA, Peters HA, Grebenchtchikov N, Look MP, Meijer-van Gelder ME, Geurts-Moespot A, et al High tumor levels of vascular endothelial growth factor predict poor response to systemic therapy in advanced breast cancer. Cancer Res. 2001;61: 5407–5414. 11454684

[48] Linderholm B, Grankvist K, Wilking N, Johansson M, Tavelin B, Henriksson R. Correlation of vascular endothelial growth factor content with recurrences, survival, and first relapse site in primary node-positive breast carcinoma after adjuvant treatment. J Clin Oncol. 2001;18: 1423–1431. 10.1200/JCO.2000.18.7.1423 10735889

[49] Liu CY, Xu JY, Shi XY, Huang W, Ruan TY, Xie P, et al M2-polarized tumor-associated macrophages promoted epithelial-mesenchymal transition in pancreatic cancer cells, partially through TLR4/IL-10 signaling pathway. Lab Invest. 2013;93: 844–854. 10.1038/labinvest.2013.69 23752129

[50] Komohara Y, Horlad H, Ohnishi K, Fujiwara Y, Bai B, Nakagawa T, et al Importance of direct macrophage-tumor cell interaction on progression of human glioma. Cancer Sci. 2012;103: 2165–2172. 10.1111/cas.12015 22957741 PMC7659278

[51] Fan QM, Jing YY, Yu GF, Kou XR, Ye F, Gao L, et al Tumor-associated macrophages promote cancer stem cell-like properties via transforming growth factor-beta1-induced epithelial-mesenchymal transition in hepatocellular carcinoma. Cancer Lett. 2014;352:160–168. 10.1016/j.canlet.2014.05.008 24892648

[52] Yang J, Liao D, Chen C, Liu Y, Chuang TH, Xiang R, et al Tumor-associated macrophages regulate murine breast cancer stem cells through a novel paracrine EGFR/Stat3/Sox-2 signaling pathway. Stem Cells. 2013;31: 248–258. 10.1002/stem.1281 23169551

[53] Upadhyay G, Chowdhury AH, Vaidyanathan B, Kim D, Saleque S. Antagonistic actions of Rcor proteins regulate LSD1 activity and cellular differentiation. Proc Natl Acad Sci U S A. 2014;111: 8071–8076. 10.1073/pnas.1404292111 24843136 PMC4050576

[54] Barrios AP, Gomez AV, Saez JE, Ciossani G, Toffolo E, Battaglioli E, et al Differential properties of transcriptional complexes formed by the CoREST family. Mol Cell Biol. 2014;34:2760–2770. 24820421 10.1128/MCB.00083-14 PMC4097654

[55] Yang P, Wang Y, Chen J, Li H, Kang L, Zhang Y, et al RCOR2 is a subunit of the LSD1 complex that regulates ESC property and substitutes for SOX2 in reprogramming somatic cells to pluripotency. Stem Cells. 2011;29: 791–801. 10.1002/stem.634 21433225

[56] Ouyang J, Shi Y, Valin A, Xuan Y, Gill G. Direct binding of CoREST1 to SUMO-2/3 contributes to gene-specific repression by the LSD1/CoREST1/HDAC complex. Mol Cell. 2009;34: 145–154. 10.1016/j.molcel.2009.03.013 19394292 PMC2727917

[57] Domanitskaya E, Schupbach T. CoREST acts as a positive regulator of Notch signaling in the follicle cells of Drosophila melanogaster. J Cell Sci. 2012;125: 399–410. 10.1242/jcs.089797 22331351 PMC3283875

[58] Kang FW, Que L, Wu M, Wang ZL, Sun J. Effects of trichostatin A on HIF-1alpha and VEGF expression in human tongue squamous cell carcinoma cells in vitro. Oncol Rep. 2012;28: 193–199. 10.3892/or.2012.1784 22552321

[59] Kashyap V, Ahmad S, Nilsson EM, Helczynski L, Kenna S, Persson JL, et al The lysine specific demethylase-1 (LSD1/KDM1A) regulates VEGF-A expression in prostate cancer. Mol Oncol. 2013;7: 555–566. 10.1016/j.molonc.2013.01.003 23384557 PMC3661758

[60] Jin L, Hanigan CL, Wu Y, Wang W, Park BH, Woster PM, et al Loss of LSD1 (lysine-specific demethylase 1) suppresses growth and alters gene expression of human colon cancer cells in a p53- and DNMT1(DNA methyltransferase 1)-independent manner. Biochem J. 2013;449: 459–468. 10.1042/BJ20121360 23072722 PMC3525012

[61] Huang Y, Stewart TM, Wu Y, Baylin SB, Marton LJ, Perkins B, et al Novel oligoamine analogues inhibit lysine-specific demethylase 1 and induce reexpression of epigenetically silenced genes. Clin Cancer Res. 2009;15: 7217–7228. 10.1158/1078-0432.CCR-09-1293 19934284 PMC2927136

[62] Wang Y, Zhang H, Chen Y, Sun Y, Yang F, Yu W, et al LSD1 is a subunit of the NuRD complex and targets the metastasis programs in breast cancer. Cell. 2009;138: 660–672. 10.1016/j.cell.2009.05.050 19703393

[63] Meier K, Mathieu EL, Finkernagel F, Reuter LM, Scharfe M, Doehlemann G, et al LINT, a novel dL(3)mbt-containing complex, represses malignant brain tumour signature genes. PLoS Genet. 2012;8: e1002676 10.1371/journal.pgen.1002676 22570633 PMC3342951

