# Supplementary figures and images for: CoREST1 Promotes Tumor Formation and Tumor Stroma Interactions in a Mouse Model of Breast Cancer
Source: PLoS One. 2015 Mar 20;10(3):e0121281. doi: 10.1371/journal.pone.0121281 (PMC4368644; doi:10.1371/journal.pone.0121281)

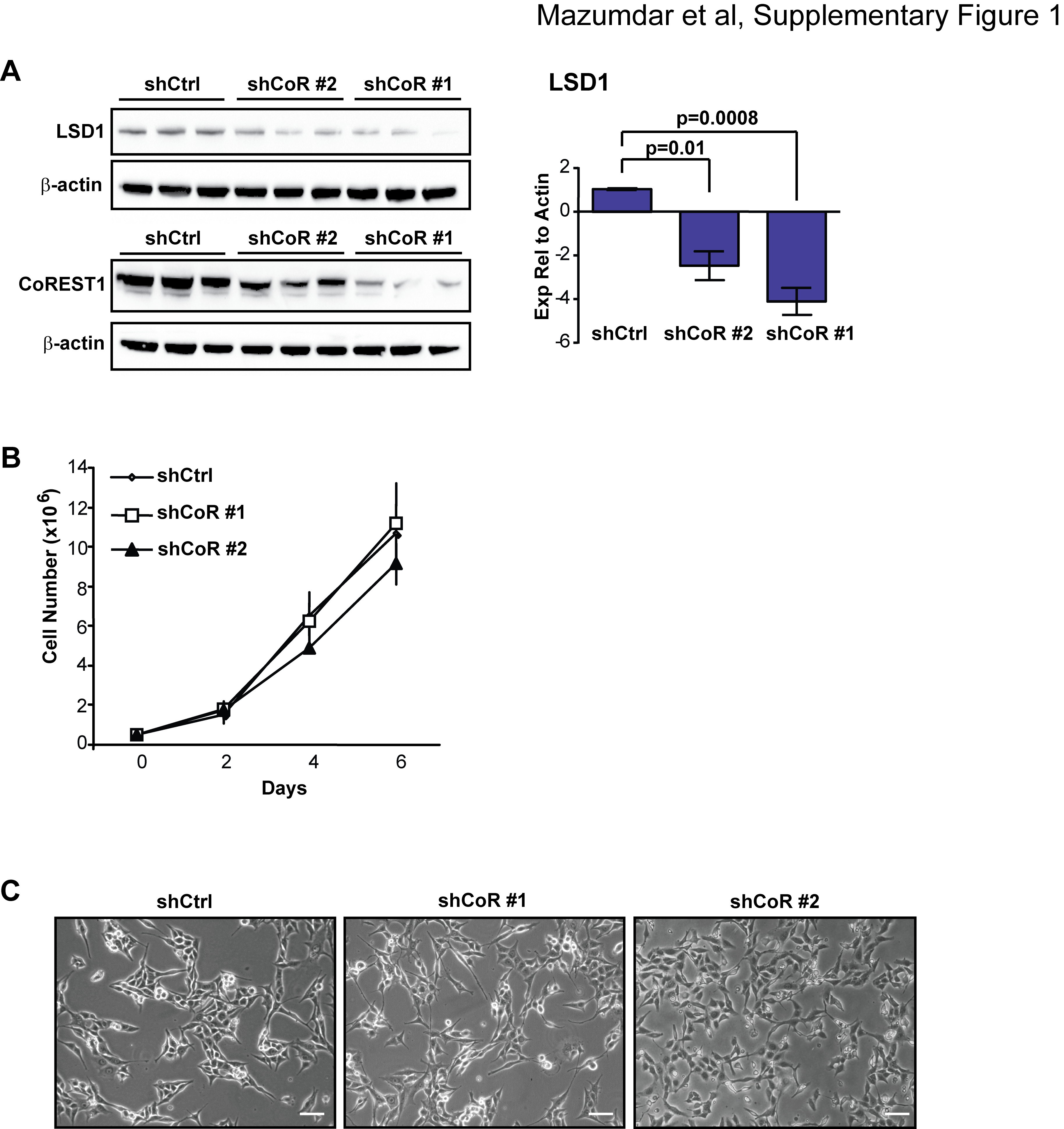

Supplement: S1 Fig — (A) Immunoblotting of LSD1 and CoREST1 in lysates from MDA-MB-231 breast cancer cells stably transfected with control (shCtrl) or either of 2 shRNA constructs targeting CoREST1 (shCoR). (B) Proliferation rates of control (shCtrl) and shCoREST1 (shCoR) MDA-MB-231 cell lines were not significantly different. Cells were plated and quantified at the indicated times as described in Materials and Methods. (C) No significant differences were observed in cell morphology in MDA-MB-231 cells with and without CoREST1 depletion. Scale bar = 100μm. (TIF) [file pone.0121281.s001.tif]

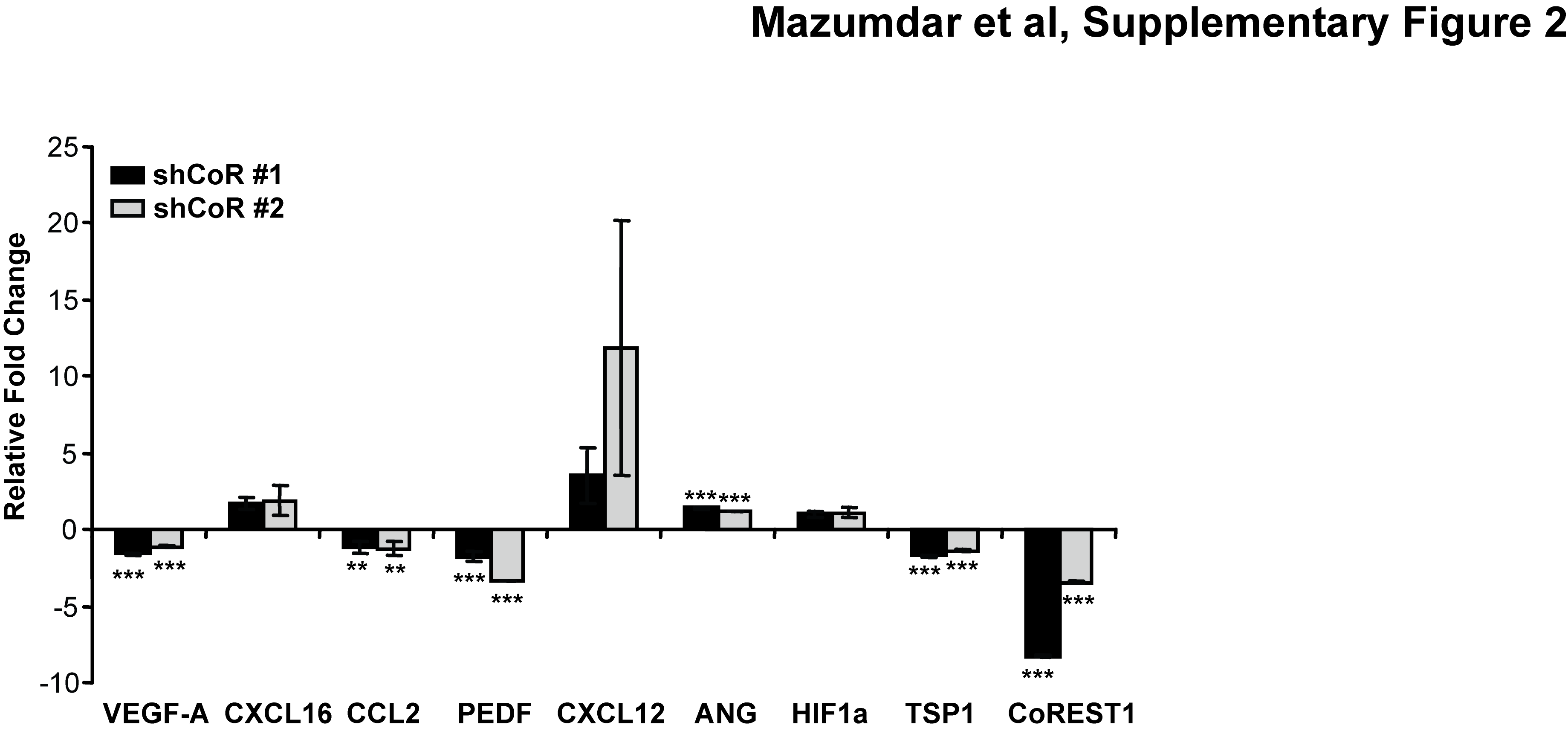

Supplement: S2 Fig — Expression levels of the indicated genes were quantified from control (shCtrl) or CoREST1 depleted (shCoR #1 and shCoR #2) MDA-MB-231 cells using RT-qPCR. Values represented as a fold change compared to shCtrl cells. (TIF) [file pone.0121281.s002.tif]
