# Supplementary material for: CoREST1 Promotes Tumor Formation and Tumor Stroma Interactions in a Mouse Model of Breast Cancer
Source: PLoS One. 2015 Mar 20;10(3):e0121281. doi: 10.1371/journal.pone.0121281 (PMC4368644; doi:10.1371/journal.pone.0121281)
Supplement: S1 Table — (DOCX) [file pone.0121281.s003.docx]

**Table S1. Primers used for qRT PCR analysis.**

|  | **Forward 5’🡪3’** | **Reverse 5’🡪3’** |
| --- | --- | --- |
| ANG | ACACTTCCTGACCCAGCACT | CCGTCTCCTCATGATGCTTT |
| CCL2 | GTCTCTGCCGCCCTTCTGT | TTGCATCTGGCTGAGCGAG |
| CoREST1 | ACTTCCAGAGGTCATTCAG | CTTCGGGCATCTTAATGG |
| CXCL12 | TGAGAGCTCGCTTTGAGTGA | CACCAGGACCTTCTGTGGAT |
| CXCL16 | AAGCCATTGAGACACCAGCTG | ACCTCGCTCTGACTCCCAGA |
| GAPDH | CGGATTTGGTCGTATTGGGC | TGGAAGATGGTGATGGGATTTC |
| HIF-1α | CAATACCCTATGTAGTTGTGGAAGTTTATG | ACCAACAGGGTAGGCAGAACATT |
| PEDF | TCCAATGCAGAGGAGTAGCA | TGTGCAGGCTTAGAGGGACT |
| TSP1 | TTGTCTTTGGAACCACACCA | CTGGACAGCTCATCACAGGA |
| VEGF-A | GCAGAATCATCACGAAGTGG | GCATGGTGATGTTGGACTCC |
