# Supplementary material for: CoREST1 Promotes Tumor Formation and Tumor Stroma Interactions in a Mouse Model of Breast Cancer
Source: PLoS One. 2015 Mar 20;10(3):e0121281. doi: 10.1371/journal.pone.0121281 (PMC4368644; doi:10.1371/journal.pone.0121281)
Supplement: S2 Table — Conditioned media from shcontrol (shCtrl) and shCoREST1 (shCoR #1) MDA-MB-231 cells was incubated with a human angiogenesis antibody array as described in Materials and Methods. Quantification of the relative pixel density for all of the factors present on the array (n = 1 experiment). (DOCX) [file pone.0121281.s004.docx]

**Table S2. Factors quantified using Human Angiogenesis Array.**

|  | Relative Pixel Density | |
| --- | --- | --- |
| **Array Factors** | **shCtrl** | **shCoR #1** |
| Activin A | nd | nd |
| ADAMTS-1 | nd | nd |
| Angiogenin (ANG) | 34.6 | 2.8 |
| Angiopoietin-1 (Ang-1) | nd | nd |
| Angiopoietin-2 (Ang-2) | nd | nd |
| Angiostatin (Plasminogen) | nd | nd |
| Amphiregulin | nd | nd |
| Artemin | nd | nd |
| Coagulation Factor III | nd | nd |
| CXCL16 | 21.7 | 1.9 |
| DPPIV (CD26) | -0.6 | 1.64 |
| EGF | nd | nd |
| EG-VEGF (PK1) | nd | nd |
| Endoglin (CD105) | nd | nd |
| Endostatin (Collagen XVIII) | nd | nd |
| Endothelin-1 (ET-1) | 13.9 | 12.9 |
| FGF-1 | nd | nd |
| FGF-2 | nd | nd |
| FGF-4 | nd | nd |
| FGF-7 | nd | nd |
| GDNF | nd | nd |
| GM-CSF | 48.3 | -1.1 |
| HB-EGF | 1.3 | 1.3 |
| HGF | nd | nd |
| IGFBP-1 | nd | nd |
| IGFBP-2 | nd | nd |
| IGFBP-3 | nd | nd |
| IL-1β | nd | nd |
| IL-8 | 39.9 | 1.2 |
| LAP (TGF-β1) | nd | nd |
| Leptin | nd | nd |
| CCL2 | 68.7 | 0.9 |
| CCL3 | nd | nd |
| MMP-8 | 8.1 | 1.9 |
| MMP-9 | 2.8 | 1.5 |
| NRG1-β1 (HRG1-β1) | nd | nd |
| Pentraxin 3 (PTX3) | nd | nd |
| PD-ECGF | nd | nd |
| PDGF-AA | nd | nd |
| PDGF-AB/PDGF-BB | nd | nd |
| Persephin | nd | nd |
| CXCL4 | nd | nd |
| PlGF | nd | nd |
| Prolactin | nd | nd |
| Maspin | nd | nd |
| PAI-1 | 1.8 | -0.7 |
| PEDF | 34.2 | 9.8 |
| TIMP-1 | 147.7 | 105.8 |
| TIMP-4 | nd | nd |
| TSP-1 | 9.2 | 0.08 |
| TSP-2 | nd | nd |
| uPA | nd | nd |
| Vasohibin | nd | nd |
| VEGF | 91.9 | 23.5 |
| VEGF-C | nd | nd |

nd = not detected
